# Increasing Ca^2+^ accumulation in salt glands under salt stress increases stronger selective secretion of Na^+^ in *Plumbago auriculata* tetraploids

**DOI:** 10.3389/fpls.2024.1376427

**Published:** 2024-04-15

**Authors:** Yifan Duan, Liqiong Jiang, Ting Lei, Keyu Ouyang, Cailei Liu, Zi’an Zhao, Yirui Li, Lijuan Yang, Jiani Li, Shouli Yi, Suping Gao

**Affiliations:** ^1^College of Landscape Architecture, Sichuan Agricultural University, Chengdu, China; ^2^Chengdu Academy of Agriculture and Forestry Sciences, Chengdu, China; ^3^College of Fine Art and Calligraphy, Sichuan Normal University, Chengdu, China

**Keywords:** recretohalophyte, salt gland, salt stress, selective secretion, tetraploid

## Abstract

Under salt stress, recretohalophyte Plumbago auriculata tetraploids enhance salt tolerance by increasing selective secretion of Na^+^ compared with that in diploids, although the mechanism is unclear. Using non-invasive micro-test technology, the effect of salt gland Ca^2+^ content on Na^+^ and K^+^ secretion were investigated in diploid and tetraploid P. auriculata under salt stress. Salt gland Ca^2+^ content and secretion rates of Na^+^ and K^+^ were higher in tetraploids than in diploids under salt stress. Addition of exogenous Ca^2+^ increased the Ca^2+^ content of the salt gland in diploids and is accompanied by an increase in the rate of Na^+^ and K^+^ secretion. With addition of a Ca^2+^ channel inhibitor, diploid salt glands retained large amounts of Ca^2+^, leading to higher Ca^2+^ content and Na^+^ secretion rate than those of tetraploids. Inhibiting H_2_O_2_ generation and H^+^-ATPase activity altered Na^+^ and K^+^ secretion rates in diploids and tetraploids under salt stress, indicating involvement in regulating Na^+^ and K^+^ secretion. Our results indicate that the increased Na^+^ secretion rate of salt gland in tetraploids under salt stress was associated with elevated Ca^2+^ content in salt gland.

## Highlight

- The Ca^2+^ content in salt glands and the rate of Na^+^ secretion were analyzed in diploid and tetraploid *P. auriculata* under salt stress, and high tetraploid rate of Na^+^ secretion was strongly correlated with high salt gland Ca^2+^ accumulation.

## 1 Introduction

Salt stress is a major abiotic stress that affects plant growth (Ruan et al., 2010). In the early stages of salt stress, high salt concentrations limit plant water uptake, resulting in osmotic stress. With prolonged salt stress, accumulation of excessive salt ions can cause oxidative stress, ion toxicity, and nutrient deficiencies in plants (Munns and Tester, 2008). Plants increase salt tolerance by various mechanisms. In nonsucculent halophytes, one particular adaptation is the secretion of excess salt ions from stem and leaf surfaces, and plants that increase salt tolerance using this pathway are called recretohalophytes (Flowers et al., 2008; Guo et al., 2023). Recretohalophytes can secrete multiple ions, and secretion rates are influenced by rhizosphere ion concentrations (Ding et al., 2010).

The mechanism of salt secretion by the salt glands is still unclear, and only possible paths have been suggested. There are three hypotheses of secretion include (1) the role of the osmotic potential in salt secretion; (2) a transfer system that is similar to liquid flow in animals; (3) salt solution excretion by vesicles in the plasma membrane (Yuan et al., 2016). Secretion rates of Na^+^ and Cl^–^ are usually higher than those of other ions (Yuan et al., 2016), which may be due to the many Na^+^ and Cl^–^ transport channels on the plasma membrane of salt gland cells. However, there are some recretohalophytes, such as those in the family Plumbaginaceae, in which Ca^2+^ is the predominant ion secreted (Faraday and Thomson, 1986; Duan et al., 2023).

The Ca^2+^ ion can be a signaling molecule in plants in response to salt stress. Exogenous addition of Ca^2+^ inhibits Na^+^ uptake by roots under salt stress and reduces K^+^ loss, thereby maintaining plant Na^+^/K^+^ ratio (Jin et al., 2017). The ion K^+^ is an essential activator of various enzymes in plant metabolic processes, and therefore, maintaining K^+^ homeostasis under salt stress is crucial to increase salt tolerance (Horie et al., 2009). The plasma membrane Na^+^/H^+^ antiporter SOS1 plays a key role in Na^+^ efflux from cells and is activated by Ca^2+^ signaling. Salt stress increases cytosolic Ca^2+^ levels, leading to the activation of SOS3, which binds to SOS2 to form the SOS3–SOS2 complex, ultimately phosphorylating and activating SOS1 for Na^+^ efflux (Qiu et al., 2002; Zhu, 2016). The SOS1 relies on the activity of plasma membrane H^+^-ATPase for ion translocation across the membrane (Chen J. A. et al., 2010; Sanadhya et al., 2015; Che et al., 2019), whereas Ca^2+^ may regulate H^+^-ATPase activity (Sun et al., 2010). Hydrogen peroxide (H_2_O_2_) can be an important signaling molecule in regulating the Na^+^/K^+^ balance. Under salt stress, H_2_O_2_ promotes Na^+^ efflux by stabilizing the mRNA of SOS1, and inhibiting H_2_O_2_ production leads to increased K^+^ efflux under salt stress (Chung et al., 2008). The Ca^2+^ ion acts as an upstream signal for H_2_O_2_, suggesting that Ca^2+^ is involved in regulating the Na^+^/K^+^ balance by modulating H_2_O_2_ production.

In recretohalophytes, Ca^2+^ is involved in regulating ion secretion from salt glands, and exogenous addition of Ca^2+^ significantly increases Na^+^ secretion under salt stress (Ding et al., 2010). Salt gland excretion of ions in recretohalophytes depends on ion transport systems and vesicular transport (Li et al., 2020). The plasma membrane Na^+^/H^+^ antiporter SOS1 is a crucial pathway for salt gland Na^+^ secretion (Guo et al., 2020), and Ca^2+^ acts as an activating signal upstream of SOS1, potentially increasing Na^+^ secretion. Addition of Ca^2+^ also increases vesicular transport, promoting Na^+^ secretion (Ding et al., 2010).

Research on the ion channels involved in K^+^ secretion of recretohalophytes is limited, and currently, K^+^ secretion is proposed to primarily occur by a Na^+^-K^+^-Cl^–^ cotransporter (Yuan et al., 2016). Under salt stress, plasma membrane depolarization in plant cells leads to activation of outward-rectifying K^+^ channels (DA-KORCs) and nonselective cation channels (DA-NSCCs), resulting in K^+^ efflux from cells (Shabala and Pottosin, 2014). However, whether DA-KORCs and DA-NSCCs are involved in K^+^ secretion in salt glands of recretohalophytes remains unclear. When exogenous Ca^2+^ channel inhibitors are added, which reduce plant Ca^2+^ levels, only the Na^+^ secretion rate is inhibited, without significant effects on K^+^ secretion rate (Kobayashi et al., 2007). Therefore, in addition to a Na^+^-K^+^-Cl^–^ cotransporter, K^+^ secretion may also occur through other ion channels.

*Plumbago auriculata* (Plumbaginaceae) is a typical calcium-secreting plant that has important medicinal value. Under normal growth conditions, Ca^2+^ is the primary ion secreted by *P. auriculata* salt glands. Under NaCl stress in our previous study, tetraploid *P. auriculata* are more salt tolerant than diploids with better ion homeostasis and less morphology damage under saline conditions. The Na^+^ secretion rate in whole leaves of tetraploid *P. auriculata* was higher than that in whole leaves of diploids. However, the Ca^2+^ content in tetraploid leaves is significantly lower than that in diploid leaves (Duan et al., 2023). Those results contradict the results of previous studies that suggest Ca^2+^ promotes Na^+^ secretion under salt stress. In animals, salt gland secretions indicate that sustained elevation of Ca^2+^ content in salt glands serves as a primary signal for secretion activity (Shuttleworth and Thompson, 1989; Shuttleworth and Hildebrandt, 1999). Therefore, it was hypothesized that *P. auriculata* Na^+^ and K^+^ secretion rates are regulated by salt gland Ca^2+^ content, not by the overall Ca^2+^ content in leaves. Thus, in this study, changes in Ca^2+^ content in the salt glands of diploid and autotetraploid *P. auriculata* were investigated by adding exogeneous Ca^2+^, inhibiting Ca^2+^ transport channels, and suppressing the generation of H_2_O_2_ regulated by Ca^2+^ signaling and H^+^-ATPase activity under NaCl stress. Effects of Ca^2+^ and downstream substances on salt gland Na^+^ and K^+^ secretion rates were also examined. The results provide a new perspective for exploring the reasons behind increased salt tolerance in polyploid recretohalophytes.

## 2 Materials and methods

### 2.1 Plant materials and treatments

Autotetraploid *Plumbago auriculata* Lam. (*2n* = 24) was induced by colchicine treatment of stem segments from diploid *P. auriculata* (*2n* = 12) in the laboratory of the Landscape Architecture Institute at Sichuan Agricultural University (Jiang et al., 2020). Both diploid and tetraploid cytotypes were obtained by tissue culture and were subcultured for 25 d. After root formation, all seedlings were transferred to a climate-controlled growth chamber. A total of 72 plants (36 diploids and 36 tetraploids) of similar size were grown for 3 months in a nutrient substrate composed of soil, vermiculite, and perlite in a 1:1:1 ratio (by volume). The plants were placed in an intelligent biochemical incubator (Ningbo Jiangnan Instrument Factory, Zhejiang, China) under controlled conditions: 25 °C/20 °C (day/night) temperature, 12-h day/12-h night photoperiod, 6,600 Lx light intensity, and 70% relative humidity. Observations on the structure and secretory components of the salt glands of diploids and tetraploids under normal growth conditions prior to salt stress provide a basic reference for analyzing ion secretion from diploid and tetraploid salt glands The sixth mature leaf, counted from the top, was selected from diploid and tetraploid plants for structural observation and analysis of salt gland secretory components.

To determine the effect of salt gland Ca^2+^ content on Na^+^ and K^+^ secretion in diploid and tetraploid plants under salt stress, plants were transferred to a hydroponic system containing 1/6th-strength Hoagland’s solution (pH 6.0 ± 0.2), with the nutrient solution refreshed at 5-d intervals. After 15 d of cultivation in the hydroponic system, diploid and tetraploid plants with similar growth were selected for six different treatments (**Table 1**). 300 mM NaCl was selected for salt stress treatment based on our preliminary experiments that it is the highest concentration at which both diploids and tetraploids will be stressed, but diploid salt gland secretion will not be inhibited because the stress is too severe. Treatments included LaCl_3_, used to inhibit Ca^2+^ channels; Na_3_VO_4_, used to inhibit H^+^-ATPase activity; and Diphenyleneiodonium chloride (DPI), used to inhibit plasma membrane NADPH oxidase. The CaCl_2_ and the inhibitors LaCl_3_, DPI, and Na_3_VO_4_ were pre-treated 20 min before the salt stress treatment. Salt stress treatment duration was 2 d, with six plants per treatment for each ploidy level.

**Table 1:** Concentration of drugs used in control and treatment groups.

	Control group	NaCl Treatment group	NaCl +CaCl_2_ Treatment group	NaCl +LaCl_3_ Treatment group	NaCl +DPI Treatment group	NaCl +Na_3_VO_4_ Treatment group
Diploid	1/6 Hoagland	1/6 Hoagland+300 mM NaCl	1/6 Hoagland+300 mM NaCl+5 mM CaCl_2_	1/6 Hoagland+300 mM NaCl+5 mM LaCl_3_	1/6 Hoagland+300 mM NaCl+0.2 mM DPI	1/6 Hoagland+300 mM NaCl+0.2 mM Na_3_VO_4_
Tetraploid	1/6 Hoagland	1/6 Hoagland+300 mM NaCl	1/6 Hoagland+300 mM NaCl+5 mM CaCl_2_	1/6 Hoagland+300 mM NaCl+5 mM LaCl_3_	1/6 Hoagland+300 mM NaCl+0.2 mM DPI	1/6 Hoagland+300 mM NaCl+0.2 mM Na_3_VO_4_

### 2.2 Leaf sample preparation for scanning electron microscopy

Leaf samples were prepared following the method described by Ding et al. (2010) with slight modifications. Fresh leaves of diploid and tetraploid *P. auriculata* were cut into small pieces of 0.5 cm × 0.5 cm from the leaf margin to the middle position of the leaf vein using a razor blade. Leaf pieces were fixed in 2.5% glutaraldehyde solution at room temperature for 24 h. After fixation, leaf samples were washed with 0.05 mM HNO_3_ to remove salt crystals on leaf surfaces, followed by rinsing with distilled water. Leaves were gently dried with absorbent paper and then dehydrated in a graded ethanol series (30%, 50%, 70%, 80%, 90%, and 95% ethanol). Dehydration time for the 30% to 90% ethanol series was 20 min, whereas it was 1 h for 95% ethanol. After dehydration, samples were dried using a critical point dryer (Leica, Nussloch, Germany). Dried samples were observed and photographed using a scanning electron microscope (SEM; Carl Zeiss, Oberkochen, **Germany**), and the area of salt glands was measured using ImageJ software. Twenty salt glands from six individual plants were measured for both diploid and tetraploid *P. auriculata*.

### 2.3 Salt gland density and area measurement

Leaf samples were prepared following the method described by Yuan et al. (2013). Fresh leaves of diploid and tetraploid *P. auriculata* were cut into small pieces as previously described. Leaf pieces (0.5 cm × 0.5 cm) were fixed in Carnoy’s fixative (ethanol:acetone = 3:1) and then washed with 0.05 mM HNO_3_ to remove salt crystals on leaf surfaces. Leaves were rinsed with distilled water, gently dried with absorbent paper, and then decolorized in 70% ethanol. Decolorized leaves were mounted with Hoyer’s solution, making temporary slides. To measure density and area of salt glands, slides were observed and photographed using a fluorescence microscope (Olympus, Tokyo, Japan) under UV excitation light (330–380 nm) at 20× magnification. Twenty different fields of view from six leaves were measured for both diploid and tetraploid *P. auriculata*.

### 2.4 Histological cross section of salt glands

Fresh leaves of diploid and tetraploid *P. auriculata* were cross-sectioned in the middle using a sharp blade. Leaf pieces (1.0 cm × 1.0 cm) were quickly fixed in formalin-aceto-alcohol (FAA) fixative at room temperature for 24 h. After fixation, leaf tissues were rinsed with distilled water for 10 min, dried with absorbent paper, and then sequentially dehydrated in a gradient series of ethanol (70%, 80%, 90%, 95%, 100%, and 100% ethanol for two times). After dehydration, tissues were cleared with xylene and then embedded in paraffin in a constant-temperature oven at 60 °C. Leaf-containing paraffin blocks were sectioned using a microtome (**Leica,** Nussloch, **Germany)**, producing 4 μm-thick sections. Sections were placed on glass slides coated with glycerol mounting medium and dried in a constant-temperature oven at 50 °C. Following dewaxing with xylene, rehydration with a gradient series of ethanol, and staining with SafraninO/Fast green, sections were dehydrated with ethanol and cleared with xylene. Last, sections were mounted with Canada balsam and air-dried at low temperature. Salt gland anatomical structures were observed and photographed using a fluorescence microscope (Olympus), and the external cuticle layer of salt glands was observed under UV excitation light (330–380 nm) using fluorescence mode. Twenty salt glands from six individual plants were observed for both diploid and tetraploid *P. auriculata*.

### 2.5 Energy dispersive X-ray analysis of salt gland secretory crystals

Salt gland secretory crystals were observed and photographed using an SEM (Carl Zeiss) equipped with an energy dispersive X-ray (EDX) system. Cross sections of mature leaf portions (0.5 cm × 0.5 cm) from the lower part of *P. auriculata* diploid and tetraploid leaves were cut with a sharp blade. Leaf sections were placed in a vacuum dryer for 1 h, and then, sections were attached to conductive adhesive tape on the SEM stage for EDX analysis of crystals secreted on leaf surfaces. Twenty salt glands from six leaves were analyzed for both diploid and tetraploid *P. auriculata*.

### 2.6 Measurement of Ca^2+^ content in salt gland cells

After 2-d treatment of diploid and tetraploid *P. auriculata*, mature leaves from the same position were collected and washed with 0.05 mM HNO_3_ to remove salt crystals on leaf surfaces. Leaves were rinsed with double-distilled water and quickly dried with absorbent paper to remove surface water. The lower epidermis of leaves was carefully torn off and placed in 0.4 mM Floura-8 Ca fluorescence probe dye solution, with addition of Pluronic F-127 to enhance fluorescence. To ensure sufficient contact between dye and leaf epidermis, a vacuum pump was used for 10 min, followed by incubation at room temperature for 40 min (avoiding light). After incubation, leaf epidermis tissues were thoroughly rinsed with double-distilled water to remove residual dye. Rinsed epidermis tissue was mounted with a fluorescence decay-resistant medium, making temporary slides. The fluorescence of Ca^2+^ in salt glands was observed with a laser scanning confocal microscope (Olympus LSCM) in the FITC channel, and fluorescence intensity was analyzed using ImageJ software. Twenty salt glands from six plants were observed for each treatment.

### 2.7 Measurement of Na^+^ and K^+^ secretion rates in salt glands

Instantaneous secretion rates of Na^+^ and K^+^ from individual salt glands on the abaxial side surface were measured using an NMT system (NMT-100SIM-YG; Younger USA LLC, Amherst, MA, USA) as described by Chen Z. et al. (2010). Leaves from the lower part of the same position of both diploid and tetraploid *P. auriculata* were collected following 2 d of salt stress treatment. The abaxial side epidermis of leaves was gently peeled off using forceps and fixed in a culture dish with the outer surface facing up. Test solutions (Na^+^: 1.0 mM NaCl, 0.1 mM KCl, 0.2 mM MES, pH 5.8; K^+^: 30 mM NaCl, 0.5 mM KCl, 0.2 mM MES, pH 5.8) were added to the culture dish, and samples were left undisturbed for 30 min before measurement. Under a microscope, a target salt gland was located, and a selective microelectrode (Na^+^: XY-SJ-Na; K^+^: XY-SJ-K; Xuyue, Beijing, China) was positioned approximately 5 μm above the outer surface of the salt gland without touching it. Each sample was measured for 5 min. Each group included nine replicates. The Na^+^ and K^+^ flux data were read directly and outputted using imFluxes v3.0 software (Xuyue, Beijing, China). Positive values represented Na^+^ and K^+^ secretion from a salt gland to the external environment, whereas negative values represented absorption from the external environment into a salt gland.

### 2.8 Data analyses

Data were analyzed using SPSS 19.0 (SPSS Inc., Chicago, IL, USA) for Windows, and all values are reported as the mean ± standard deviation (SD). Two-way ANOVA and Student’s *t*-test were used to compare means of different treatments for each data set at the significance level of *P* < 0.05.

## 3 Results

### 3.1 Influence of polyploidization on salt glands of *Plumbago auriculata*

**Figure 1:**
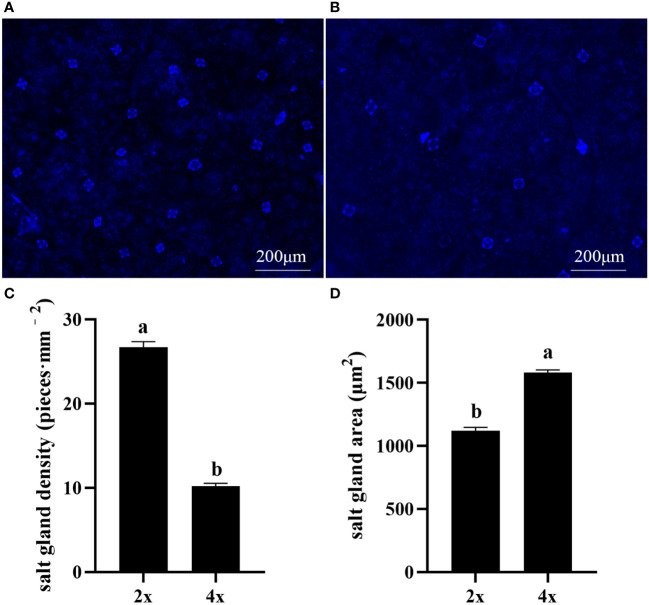
Salt gland density and salt gland area in diploid (2x) and tetraploid (4x) *Plumbago auriculata.* **(A)** Diploid salt glands and **(B)** tetraploid salt glands. **(C)** Salt gland density and **(D)** salt gland area of single salt gland in diploids and tetraploids. Different lowercase letters indicate significant differences (P < 0.05) between diploids and tetraploids.

**Figure 2:**
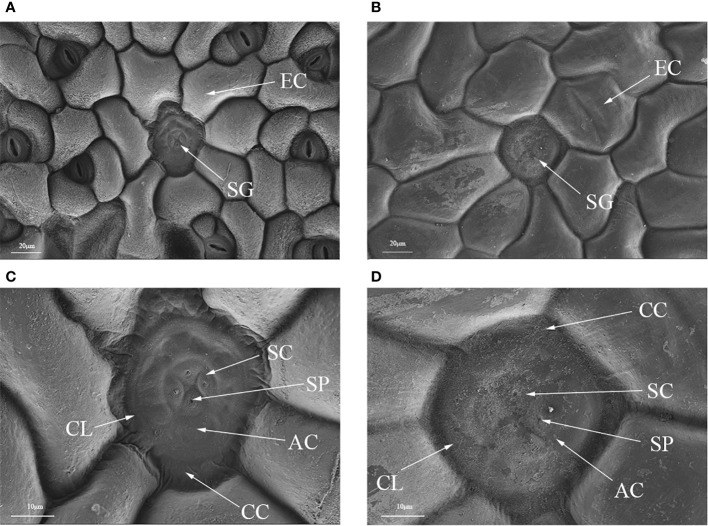
Optical microscope and scanning electron microscope images of salt glands in diploid and tetraploid *Plumbago auriculata.* **(A)** Diploid salt gland and **(B)** tetraploid salt gland observed under an optical microscope. **(C)** Diploid salt gland and **(D)** tetraploid salt gland observed under a scanning electron microscope. SG, salt gland; EP, epidermal cell; SC, secretory cell; AC, accessory cell; SP, secretion pore; CC, collecting cell; CL, cuticle layer.

**Figure 3:**
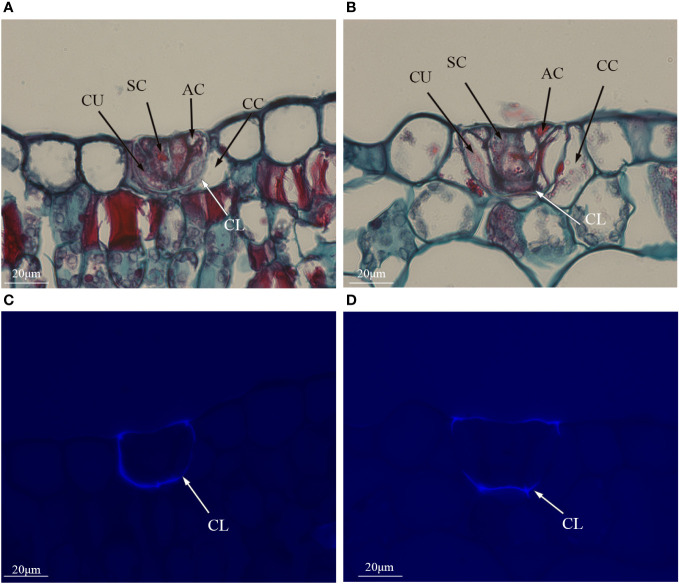
Histological cross sections of salt glands in diploid and tetraploid *Plumbago auriculata.* Cross section of **(A)** diploid salt gland and **(B)** tetraploid salt gland. Fluorescence of the cuticle layer in **(C)** diploid salt gland and **(D)** tetraploid salt gland. SC, secretory cell; AC, accessory cell; CC, collecting cell; CU, cup cell; CL, cuticle layer.

Polyploidization significantly increased salt gland area of *P. auriculata*, with the area of tetraploid salt glands 41.1% larger than that of diploid salt glands. However, salt gland density decreased significantly in tetraploid plants (**Figure 1**). There were no significant differences in salt gland structure between diploid and tetraploid *P. auriculata* (**Figures 2**, **3**). Salt glands in both cytotypes consisted of 16 salt gland cells, with four collecting cells, four accessory cells, four cup cells, and four secretory cells, each with a secretion pore in the center.

**Figure 4:**
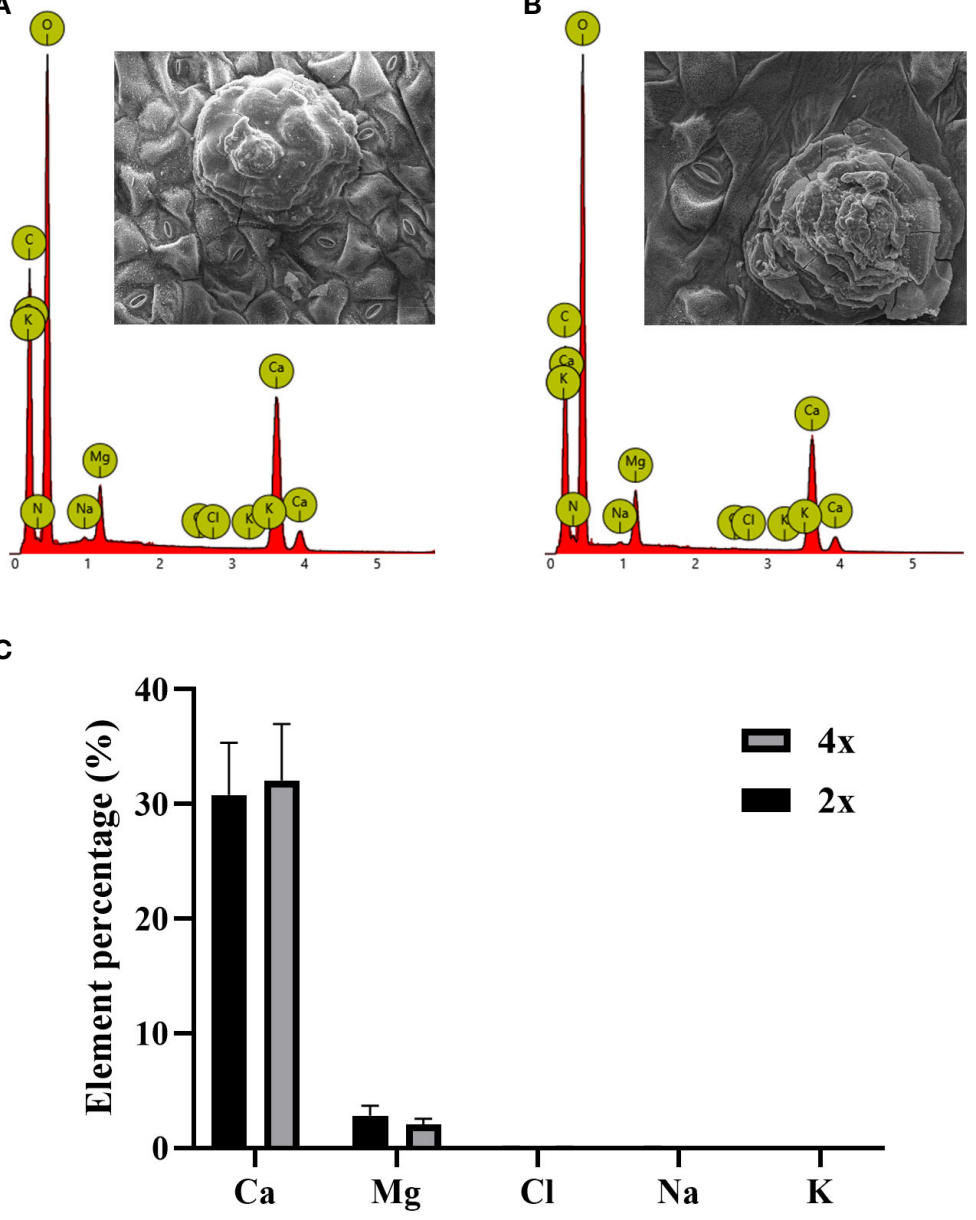
Ion composition of crystals secreted by salt glands in diploid and tetraploid *Plumbago auriculata.* Energy dispersive spectroscopy scan of secreted crystals from **(A)** diploid and **(B)** tetraploid salt glands. Element percentage of crystals secreted from the salt glands of **(C)** diploids (2x) and tetraploids (4x).

In SEM images of secreted salt gland crystals, the volume of salt crystals secreted on leaf surfaces was significantly larger in tetraploids than in diploids, indicating higher secretion capacity of individual salt glands in tetraploids under normal growth conditions. According to the EDX analysis of secreted crystals in diploids and tetraploids, the main ion secreted was Ca^2+^, followed by Mg^2+^, whereas the secretion of Na^+^ and Cl^–^ was minimal (**Figure 4**). This result suggested that polyploidization of *P. auriculata* did not significantly affect composition of salt gland secretions.

### 3.2 Polyploidization of *Plumbago auriculata* results in significant differences in Ca^2+^ content of salt glands compared with that in diploids

**Figure 5:**
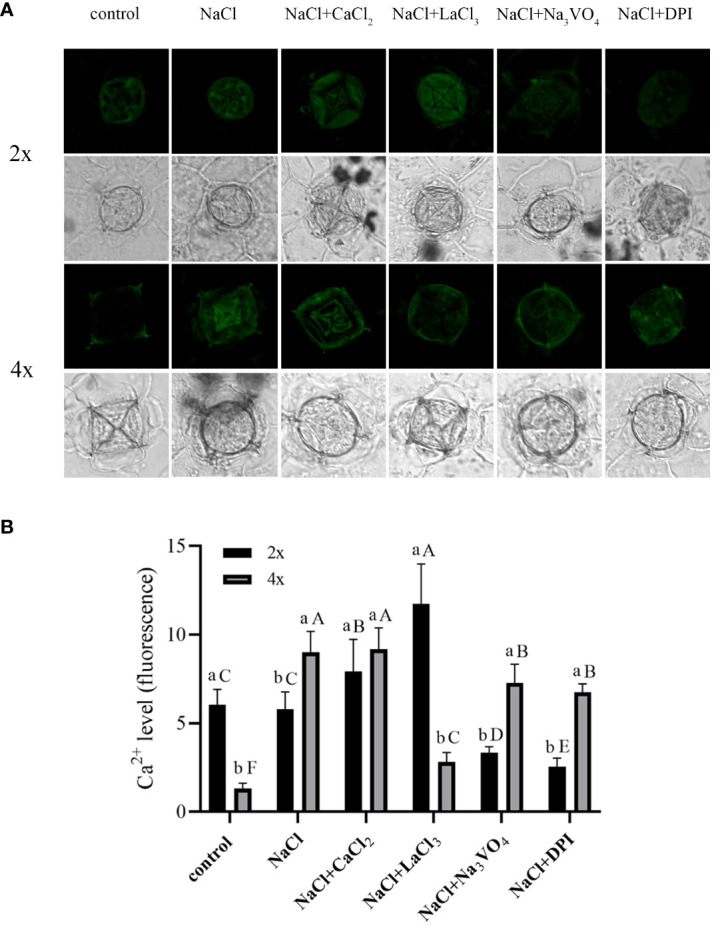
Changes in Ca^2+^ accumulation in salt glands of diploid (2x) and tetraploid (4x) *Plumbago auriculata* induced by NaCl stress and effects of LaCl_3_, Na_3_VO_4_, and DPI on Ca^2+^ accumulation under NaCl stress. **(A)** Representative graphs showing changes in Ca^2+^ accumulation in (top) diploid and (bottom) tetraploid salt glands before and after salt stress, as well as effects under LaCl_3_, Na_3_VO_4_, and DPI treatments. **(B)** Fluorescence intensity values from **(A)** measured by Image J software. For each treatment, nine salt glands from six individual plants were observed and quantified. In **(B)**, different capital letters indicate significant differences (*P* < 0.05) between different treatments of the same ploidy level, whereas different lowercase letters indicate significant differences (*P* < 0.05) between ploidy levels of the same treatment.

Under control conditions, Ca^2+^ content in salt glands of diploids was significantly higher than that in salt glands of tetraploids, with content in diploids 4.5 times higher (**Figure 5**). Compared with the control, NaCl stress significantly increased Ca^2+^ accumulation in the salt glands of tetraploid plants, whereas it did not significantly affect Ca^2+^ accumulation in diploid salt glands. After adding CaCl_2_, Ca^2+^ content in the salt glands of diploids under NaCl stress increased significantly compared with that in the NaCl treatment alone, whereas no significant change was observed in tetraploids, and then, there was no significant difference in Ca^2+^ content between diploid and tetraploid salt glands. The addition of the Ca^2+^ inhibitor LaCl_3_ suppressed NaCl-induced accumulation of Ca^2+^ in tetraploid salt glands, resulting in no significant difference in Ca^2+^ accumulation compared with the control. Notably, Ca2+ content in the salt glands of diploids increased significantly with LaCl_3_ treatment, surpassing that of both the NaCl treatment alone and tetraploid salt glands treated with LaCl_3_. When the H_2_O_2_ inhibitor DPI or the H^+^-ATPase inhibitor Na_3_VO_4_ was added, Ca^2+^ content in the salt glands of both diploids and tetraploids decreased significantly compared with that in the NaCl treatment alone.

### 3.3 Polyploidization increases Na^+^ secretion rate in salt glands under salt stress, which is significantly inhibited by the Ca^2+^ channel inhibitor LaCl_3_

**Figure 6:**
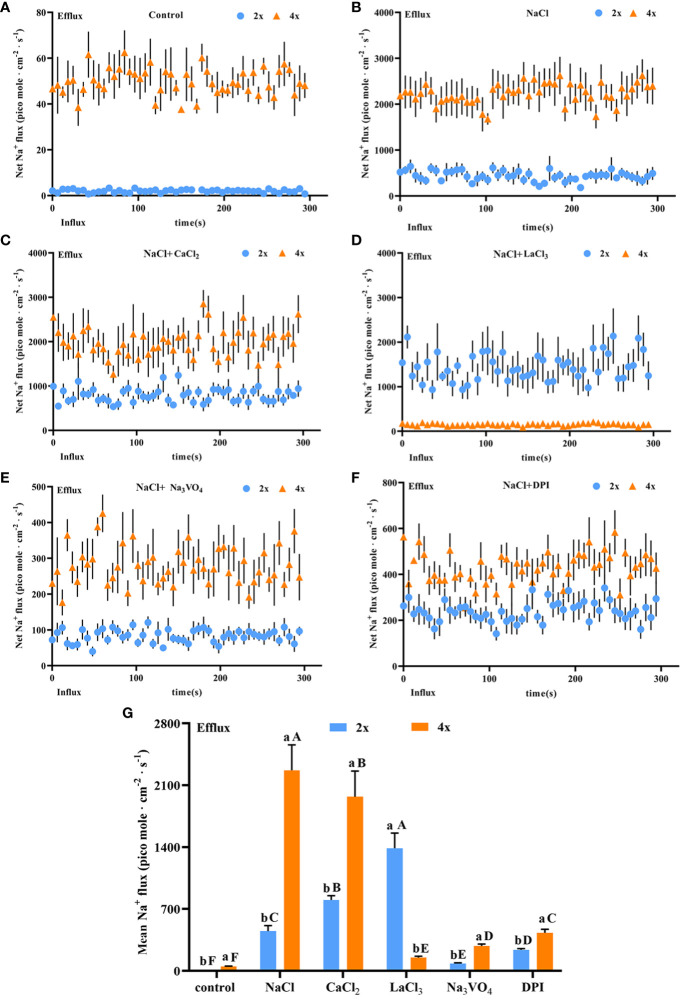
Changes in Na^+^ instantaneous and average secretion rates in salt glands of diploid (2x) and tetraploid (4x) *Plumbago auriculata* under NaCl stress and effects of LaCl_3_, Na_3_VO_4_, and DPI on Na^+^ secretion rate under NaCl stress. **(A)** Instantaneous Na^+^ secretion rate in salt glands under control conditions. **(B)** Instantaneous Na^+^ secretion rate in salt glands under NaCl stress. **(C)** Instantaneous Na^+^ secretion rate in salt glands under NaCl stress with exogenous CaCl_2_ addition. **(D)** Instantaneous Na^+^ secretion rate in salt glands under NaCl stress with LaCl_3_ addition. **(E)** Instantaneous Na^+^ secretion rate in salt glands under NaCl stress with Na_3_VO_4_ addition. **(F)** Instantaneous Na^+^ secretion rate in salt glands under NaCl stress with DPI addition. **(G)** Average Na^+^ secretion rate (~300 s) in different treatments. Each point represents the mean of the nine salt glands collected from six individual plants.

Salt stress induces strong and stable Na^+^ secretion. After salt stress, Na^+^ secretion rate increased significantly in both diploids and tetraploids and was 212.11 and 50.92 times higher, respectively, than that in the control. The Na^+^ secretion rate was 5.28 times higher in tetraploids than in diploids (**Figures 6A, B**). Thus, salt stress activated Na^+^ secretion in *P. auriculata*, and Na^+^ secretion capacity in individual salt glands under salt stress was greater in tetraploids than in diploids. Addition of CaCl_2_ significantly increased Na^+^ secretion in diploid salt glands under salt stress, with secretion 1.8 times higher than that in the NaCl treatment alone. However, CaCl_2_ addition significantly inhibited Na^+^ secretion in tetraploid salt glands, reaching 67.56% of the NaCl treatment alone. Nevertheless, the Na^+^ secretion rate in tetraploid salt glands remained significantly higher than that in diploid salt glands (**Figure 6C**). After addition of the Ca^2+^ channel inhibitor LaCl_3_, Na^+^ secretion in tetraploids was severely inhibited, reaching only 6.5% and 9.68% of that in NaCl and CaCl_2_ treatments, respectively, and with secretion only 3.26 times higher than that in the control. By contrast, the Na^+^ secretion rate in diploid salt glands increased significantly and was 2.60 and 1.44 times higher than that in NaCl and CaCl_2_ treatments, respectively, and was 7.25 times higher than that in tetraploid salt glands under the same conditions (**Figure 6D**). Pretreatment with the H^+^-ATPase inhibitor (Na_3_VO_4_) or the H_2_O_2_ inhibitor (DPI) significantly decreased Na^+^ secretion rate under salt stress in both diploids and tetraploids, indicating the important roles of endogenous H^+^-ATPase and H_2_O_2_ in increasing Na^+^ secretion rate under salt stress (**Figures 6E, F**). Overall, the Na^+^ secretion rate in tetraploids was lower than that in diploids only in the LaCl_3_+NaCl treatment, whereas in other treatments, the Na^+^ secretion rate was higher in tetraploids than in diploids (**Figure 6G**).

### 3.4 Secretion rates of K^+^ in salt glands of diploid and tetraploid Plumbago auriculata under different stress treatments

**Figure 7:**
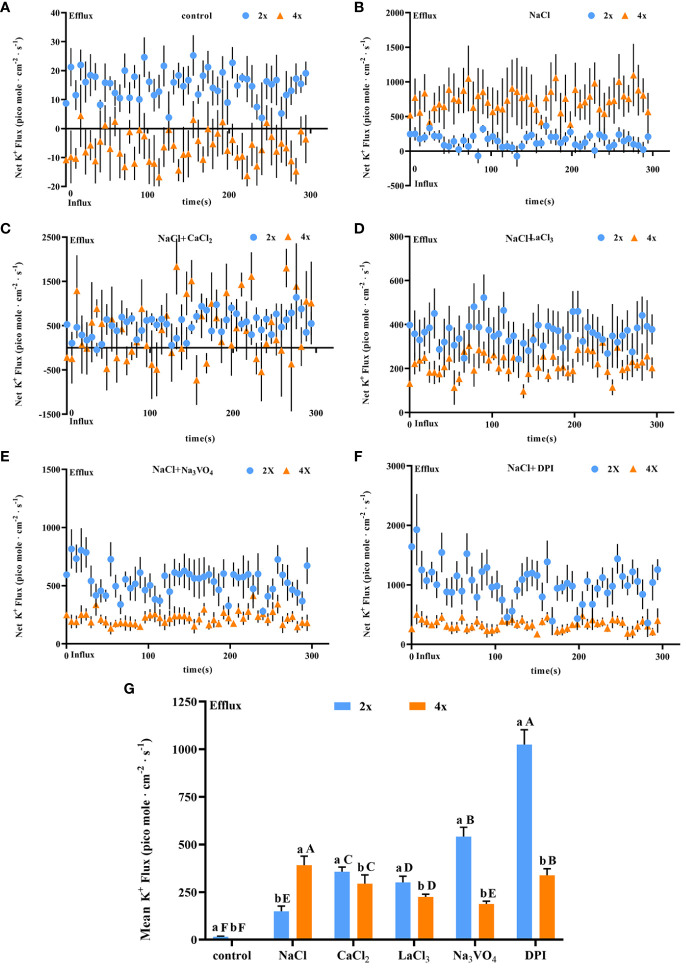
Changes in K^+^ instantaneous and average secretion rates in salt glands of diploid (2x) and tetraploid (4x) *Plumbago auriculata* induced by NaCl stress and effects of LaCl_3_, Na_3_VO_4_, and DPI on K^+^ secretion rate under NaCl stress. **(A)** Instantaneous K^+^ secretion rate in salt glands under control conditions. **(B)** Instantaneous K^+^ secretion rate in salt glands under NaCl stress. **(C)** Instantaneous K^+^ secretion rate in salt glands under NaCl stress with exogenous CaCl_2_ addition. **(D)** Instantaneous K^+^ secretion rate in salt glands under NaCl stress with LaCl_3_ addition. **(E)** Instantaneous K^+^ secretion rate in salt glands under NaCl stress with Na_3_VO_4_ addition. **(F)** Instantaneous K^+^ secretion rate in salt glands under NaCl stress with DPI addition. **(G)** Average K^+^ secretion rate (~300 s) in different treatments. Each point represents the mean of the nine salt glands collected from six individual plants.

Salt stress induced a significant increase in K^+^ secretion rate in individual salt glands of both diploids and tetraploids, with the K^+^ secretion rate in tetraploids significantly higher than that in diploids by 3.20 times. However, the Na^+^/K^+^ secretion rate ratio was also higher in tetraploids, indicating stronger selective secretion of Na^+^ in tetraploids than in diploids under salt stress (**Figures 7A, B**). After addition of CaCl_2_, K^+^ secretion in tetraploid salt glands under salt stress was inhibited, reaching 60.52% of that in the NaCl treatment, whereas K^+^ secretion in diploid salt glands increased significantly and was 3.51 times higher than that in the NaCl treatment (**Figure 7C**).

The K^+^ secretion rate was inhibited in tetraploid plants by the addition of LaCl_3_ compared with NaCl alone due to the inhibitory effect of LaCl_3_ on K^+^ channels (Wegner et al., 1994). However, it increased in diploid plants. This may be due to the fact that in addition to the inhibitory effect of LaCl_3_ on K^+^ channels the presence of large amounts of retained Ca^2+^ in the salt glands increased the overall secretion level including the K^+^ secretion rate (**Figure 7D**). Notably, the addition of the H^+^-ATPase inhibitor (Na_3_VO_4_) or the H_2_O_2_ inhibitor (DPI) inhibited K^+^ secretion in tetraploid salt glands under salt stress, whereas in diploid salt glands, secretion increased significantly (**Figures 7E, F**). Overall, the K^+^ secretion rate in tetraploid salt glands was higher than that in diploid salt glands only in the NaCl treatment, and in other treatments, the rate was lower in tetraploids than in diploids (**Figure 7G**).

## 4 Discussion

### 4.1 Polyploidization promotes secretion of individual salt glands but does not alter components of secretions under normal growth conditions

There may be a positive correlation between salt gland size and secretion rate (Feng et al., 2014; Mi et al., 2021). Polyploidization in *P. auriculata* significantly increased the size of salt glands, and under normal growth conditions, the volume of secreted crystals also increased significantly. It is hypothesized that polyploidization enhances the secretory capacity of individual salt glands of *P. auriculata* under normal growth conditions.

### 4.2 Polyploidization increases efficiency of Ca^2+^ allocation and increases its transport to salt glands under salt stress

Under control conditions, Ca^2+^ content in tetraploid salt glands was only 22.22% of that in diploid glands, indicating that tetraploids decreased the allocation of Ca^2+^ to salt glands and increased that to leaf mesophyll cells for plant growth and metabolism (Dayod et al., 2010; Gilliham et al., 2011; Nomura and Shiina, 2014). In a previous study, under control conditions, *P. auriculata* had significantly lower Ca^2+^ secretion rates in tetraploid leaves than in diploid leaves, only reaching 33% of the diploid secretion (Duan et al., 2023). Therefore, the decrease in Ca^2+^ allocation to salt glands might be an important factor contributing to the decrease in Ca^2+^ secretion rate in tetraploids compared with that in diploids under control conditions. Salt stress stimulates Ca^2+^ influx into cells (Dong et al., 2022) but also inhibits root Ca^2+^ uptake (Guo et al., 2022), leading to a decrease in overall Ca^2+^ content in plants (Guo et al., 2015). However, although Ca^2+^ content showed no significant change in diploid salt glands under salt stress, it increased significantly in tetraploid salt glands, indicating that tetraploids significantly increased Ca^2+^ transport to salt glands.

With loss of Ca^2+^, selective transport of Ca^2+^ to salt glands requires increases in energy consumption (Dassanayake and Larkin, 2017). Under control conditions, tetraploids decreased the allocation of Ca^2+^ to salt glands, thereby reducing the energy expenditure for transport and retaining more Ca^2+^ in leaf mesophyll cells as essential nutrients and signaling molecules for normal plant growth and metabolism (Dayod et al., 2010). Under salt stress, Ca^2+^ promotes Na^+^ secretion (Mahajan et al., 2008; Guo et al., 2009), and increased allocation of Ca^2+^ to salt glands might be the reason for higher Na^+^ secretion rate in tetraploids (Duan et al., 2023).

Ca^2+^ content in salt glands is affected by both Ca^2+^ transport from the chloroplasts and Ca^2+^ secretion from the salt glands. No direct evidence has been obtained to confirm the pathway of salt transport into the salt gland, but ion carriers or channels, plasmodesmata and vesicles may play an important role in transporting (Yuan et al., 2016). Ca^2+^ influx in plant cells is predominantly mediated by NSCC, and it has been shown that Ca^2+^ channel inhibitors do not only block Ca^2+^ influx but also Ca^2+^ efflux (Zherelova et al., 1994), and La^3+^ can also block outward current (Terry et al., 1992). The diploid salt glands secreted large amounts of Ca^2+^ under control conditions, and the addition of LaCl_3_ resulted in a significant increase in the Ca^2+^ content of the salt glands, probably due to the inhibition of Ca^2+^ secretion leading to its accumulation in the salt glands. At this time, we found that the Ca^2+^ content in diploid salt glands was significantly higher than that in tetraploids, and the rate of Na^+^ secretion from salt glands was also significantly higher than that in tetraploids, so we hypothesized that the Ca^2+^ content of salt glands was one of the important factors affecting the rate of Na^+^ secretion.

### 4.3 Increased Ca^2+^ content in salt glands increases Na^+^ secretion rate in *Plumbago auriculata* under salt stress

It was shown that Ca^2+^ promotes the secretion of Na^+^ from salt glands of recretohalophytes (Ding et al., 2010; Maeda, 2019). In our previous study on whole-leaf secretion rates in *P. auriculata* under salt stress, Ca^2+^ content in diploid leaves was higher than that in tetraploid leaves, but the Na^+^ secretion rate was lower in diploids than in tetraploids (Duan et al., 2023). Besides, it has been suggested in the other research that K^+^ in the salt gland promotes salt gland NaCl secretion (Feng et al., 2015). After salt stress, Ca^2+^ content in diploid salt glands was significantly lower than that in tetraploid salt glands, and the Na^+^ secretion rate in individual diploid salt glands was also significantly lower. Therefore, it was hypothesized that Ca^2+^ content in salt glands is a key factor determining salt gland Na^+^ secretion rate, not the overall Ca^2+^ content in leaves. Besides, increasing Na^+^ secrete rate is companied with increased Ca^2+^ content in salt gland of tetraploid. The plasma membrane Na^+^/H^+^ antiporter (SOS1) been shown to be involved in Na^+^ secretion in recretohalophyte with *SOS1* genes up-regulated after salt treatment and Na^+^ secretion rate was decreased when Silencing *SOS1* (Guo et al., 2020; Zhang et al., 2017). The higher secretion rate in tetraploid in salt glands under salt stress might suggested higher *SOS1*gene expression, and the molecular mechanisms require further research.

Notably, although addition of CaCl_2_ did not significantly affect Ca^2+^ content in tetraploid salt glands, the Na^+^ secretion rate in tetraploids was significantly lower than that in the NaCl treatment alone. It was presumed that because Ca^2+^ content of salt glands did not increase, the decrease in Na^+^ secretion from salt glands was due to CaCl_2_ reducing the activation of Na^+^ secretion by NaCl stress (Mulaudzi et al., 2020).

The Ca^2+^ ion is an important secondary messenger that regulates H^+^-ATPase activity and H_2_O_2_ generation (Demidchik and Shabala, 2018; Shabala, 2019). In the present study, we found that inhibition of H^+^-ATPase activity or H_2_O_2_ production inhibited both diploid and tetraploid Na^+^ secretion rates in *P. auriculata*. Ca^2+^ may also indirectly regulate Na^+^ secretion through H^+^-ATPase and H_2_O_2_ (Kong et al., 2016; Wang et al., 2020), and the mechanisms need to be further investigated.

### 4.4 K^+^ efflux channels induced by plasma membrane depolarization might participate in K^+^ secretion in *Plumbago auriculata*

Although the mechanism of salt secretion in recretohalophytes remains not fully understood, salt secretion is an active transport process that requires H^+^-ATPase to provide energy (Chen JA et al., 2010). Therefore, inhibiting H^+^-ATPase activity significantly suppresses ion secretion in salt glands. In this study, inhibiting H^+^-ATPase significantly inhibited Na^+^ secretion in both diploids and tetraploids. Notably, under salt stress, inhibiting H^+^-ATPase activity suppressed K^+^ secretion in tetraploids but significantly increased it in diploids. Therefore, it was hypothesized that the K^+^ efflux channels DA-KORCs and DA-NSCCs, activated by plasma membrane depolarization, were involved in K^+^ secretion in *P. auriculata* salt glands (Shabala et al., 2006; Sun et al., 2009). Therefore, inhibiting H^+^-ATPase activity induced severe depolarization of the plasma membrane in diploid salt gland cells, activating DA-KORCs and DA-NSCCs efflux channels (Goncalves et al., 2000) and increasing the rate of K^+^ secretion. Additionally, Ca^2+^ inhibits the activation of the K^+^ efflux channels DA-KORCs and DA-NSCCs by depolarization, and the decrease in Ca^2+^ content in salt glands after H^+^-ATPase inhibition contributed to the increase in K^+^ efflux and increased K^+^ secretion. Hydrogen peroxide is a signaling molecule that increases H^+^-ATPase activity (Lu et al., 2013), and the addition of the NADPH oxidase inhibitor DPI, which inhibits H_2_O_2_ production, increases the loss of K^+^ caused by depolarization of the plasma membrane (Ma et al., 2012). By contrast, the secretion of K^+^ was inhibited in tetraploids following inhibition of H^+^-ATPase or H_2_O_2_, suggesting that the effect of depolarization-induced K^+^ efflux on tetraploids was relatively minor than diploids.

## 5 Conclusion

Rates of Na^+^ and K^+^ secretion in salt glands of the recretohalophyte *P. auriculata* were regulated by Ca^2+^ content in the glands. The high Ca^2+^ content in salt glands was an important factor contributing to higher Na^+^ secretion rates in tetraploids than in diploids under salt stress. The downstream signals of Ca^2+^, H_2_O_2_ and H^+^-ATPase, were also involved in regulating Na^+^ and K^+^ secretion rates. In addition to directly promoting ion secretion, Ca^2+^ might have indirectly regulated Na^+^ and K^+^ secretion by modulating H_2_O_2_ and H^+^-ATPase. Inhibiting H_2_O_2_ production or H^+^-ATPase activity significantly increased the rate of K^+^ secretion in diploids under salt stress, suggesting the involvement of depolarization-activated K^+^ efflux channels (DA-KORCs and DA-NSCCs) in salt stress-induced salt gland K^+^ secretion. Overall, polyploidization in *P. auriculata* led to an increase in utilization efficiency of Ca^2+^, resulting in increased accumulation of Ca^2+^ in salt glands under salt stress. This, in turn, increased Na^+^ secretion from salt glands and decreased Na^+^ accumulation in plant tissues, thereby mitigating the damage caused by salt stress.

## Data availability statement

The original contributions presented in the study are included in the article/supplementary material. Further inquiries can be directed to the corresponding author.

## Author contributions

YD: Writing – original draft, Writing – review & editing. LJ: Funding acquisition, Writing – review & editing. TL: Writing – review & editing. KO: Writing – review & editing. CL: Writing – review & editing. ZZ: Writing – review & editing. YL: Writing – review & editing. LY: Writing – review & editing. JL: Writing – review & editing. SY: Writing – review & editing. SG: Writing – review & editing.

## References

[B1] Che B. Cheng C. Fang J. Liu Y. Yu B. (2019). The recretohalophyte *Tamarix TrSOS1* gene confers enhanced salt tolerance to transgenic hairy root composite cotton seedlings exhibiting virus-induced gene silencing of *GhSOS1* . Int. J. Mol. Sci. 20, 2930. doi: 10.3390/ijms20122930 31208046 PMC6628528

[B2] Chen Z. Newman I. Zhou M. Mendham N. Zhang G. Shabala S. (2010). Screening plants for salt tolerance by measuring K^+^ flux: a case study for barley. Plant Cell Environ. 28, 1230–1246. doi: 10.1111/j.1365-3040.2005.01364.x

[B3] Chen J. A. Xiao Q. A. Wu F. H. Dong X. J. He J. X. Pei Z. M.. (2010). Nitric oxide enhances salt secretion and Na^+^ sequestration in a mangrove plant, Avicennia marina, through increasing the expression of H^+^-ATPase and Na^+^/H^+^ antiporter under high salinity. Tree Physiol. 30, 1570–1585. doi: 10.1093/treephys/tpq086 21030403

[B4] Chung J. S. Zhu J. K. Bressan R. A. Hasegawa P. M. Shi H. H. (2008). Reactive oxygen species mediate Na^+^-induced *SOS1* mRNA stability in *Arabidopsis* . Plant J. 53, 554–565. doi: 10.1111/j.1365-313X.2007.03364.x 17996020 PMC3128381

[B5] Dassanayake M. Larkin J. C. (2017). Making plants break a sweat: the structure, function, and evolution of plant salt glands. Front. Plant Sci. 8. doi: 10.3389/fpls.2017.00406 PMC5368257 28400779

[B6] Dayod M. Tyerman S. D. Leigh R. A. Gilliham M. (2010). Calcium storage in plants and the implications for calcium biofortification. Protoplasma 247, 215–231. doi: 10.1007/s00709-010-0182-0 20658253

[B7] Demidchik V. Shabala S. (2018). Mechanisms of cytosolic calcium elevation in plants: the role of ion channels, calcium extrusion systems and NADPH oxidase-mediated ‘ROS-Ca^2+^ Hub’. Funct. Plant Biol. 45, 9–27. doi: 10.1071/FP16420 32291018

[B8] Ding F. Chen M. Sui N. Wang B. S. (2010). Ca^2+^ significantly enhanced development and salt-secretion rate of salt glands of *Limonium bicolor* under NaCl treatment. South Afr. J. Botany. 76, 95–101. doi: 10.1016/j.sajb.2009.09.001

[B9] Dong Q. Y. Wallrad L. Almutairi B. O. Kudla J. (2022). Ca^2+^ signaling in plant responses to abiotic stresses. J. Integr. Plant Biol. 64, 287–300. doi: 10.1111/jipb.13228 35048537

[B10] Duan Y. F. Lei T. Li W. J. Jiang M. Y. Zhao Z. A. Yu X. F.. (2023). Enhanced Na^+^ and Cl^-^ sequestration and secretion selectivity contribute to high salt tolerance in the tetraploid recretohalophyte *Plumbago auriculata* Lam. Planta 257 (3), 52. doi: 10.1007/s00425-023-04082-7 36757459

[B11] Faraday C. D. Thomson W. W. (1986). Functional aspects of the salt glands of the Plumbaginaceae. J. Exp. Botany. 37, 1129–1135. doi: 10.1093/jxb/37.8.1129

[B12] Feng Z. T. Sun Q. J. Deng Y. Q. Sun S. F. Zhang J. G. Wang B. S. (2014). Study on pathway and characteristics of ion secretion of salt glands of *Limonium bicolor* . Acta Physiologiae Plantarum 36, 2729–2741. doi: 10.1007/s11738-014-1644-3

[B13] Feng Z. T. Deng Y. Q. Zhang S. C. Liang X. Yuan F. Hao J. L.. (2015). K^+^ accumulation in the cytoplasm and nucleus of the salt gland cells of *Limonium bicolor* accompanies increased rates of salt secretion under NaCl treatment using NanoSIMS. Plant Science 238, 286–296. doi: 10.1016/j.plantsci.2015.06.021 26259195

[B14] Flowers T. J. Colmer T. D. (2008). Salinity tolerance in halophytes. New Phytol. 179, 945–963. doi: 10.1111/j.1469-8137.2008.02531.x 18565144

[B15] Gilliham M. Dayod M. Hocking B. J. Xu B. Conn S. J. Kaiser B. N.. (2011). Calcium delivery and storage in plant leaves: exploring the link with water flow. J. Exp. Botany. 62, 22–50. doi: 10.1093/jxb/err111 21511913

[B16] Goncalves P. P. Meireles S. M. Neves P. Vale M. G. (2000). Distinction between Ca^2+^ pump and Ca^2+^/H^+^ antiport activities in synaptic vesicles of sheep brain cortex. Neurochemist. Int. 37, 387–396. doi: 10.1016/S0197-0186(00)00009-7 10825579

[B17] Guo K. M. Babourina O. Rengel Z. (2009). Na^+^/H^+^ antiporter activity of the *SOS1* gene: lifetime imaging analysis and electrophysiological studies on *Arabidopsis* seedlings. Plant Physiol. 137, 155–165. doi: 10.1111/j.1399-3054.2009.01274.x 19758408

[B18] Guo J. X. Lu X. Y. Tao Y. F. Guo H. J. Min W. (2022). Comparative ionomics and metabolic responses and adaptive strategies of cotton to salt and alkali stress. Front. Plant Sci. 13. doi: 10.3389/fpls.2022.871387 PMC9084190 35548284

[B19] Guo Q. Meng L. Han J. W. Mao P. C. Tian X. X. Zheng M. L.. (2020). SOS1 is a key systemic regulator of salt secretion and K^+^/Na^+^ homeostasis in the recretohalophyte *Karelinia caspia* . Environ. Exp. Bot. 177, 104098. doi: 10.1016/j.envexpbot.2020.104098

[B20] Guo Z. Wei M. Y. Zhong Y. H. Wu X. Chi B. J. Li J.. (2023). Leaf sodium homeostasis controlled by salt gland is associated with salt tolerance in mangrove plant *Avicennia marina* . BMC Plant Biol. 43, 817–831. doi: 10.1093/treephys/tpad002 36611000

[B21] Guo R. Yang Z. Z. Li F. Yan C. R. Zhong X. L. Liu Q.. (2015). Comparative metabolic responses and adaptive strategies of wheat (*Triticum aestivum*) to salt and alkali stress. BMC Plant Biol. 15, 170. doi: 10.1186/s12870-015-0546-x 26149720 PMC4492011

[B22] Horie T. Hauser F. Schroeder J. I. (2009). HKT transporter-mediated salinity resistance mechanisms in *Arabidopsis* and monocot crop plants. Trends Plant Sci. 12, 660–668. doi: 10.1016/j.tplants.2009.08.009 PMC2787891 19783197

[B23] Jiang Y. L. Liu S. L. Hu J. He G. T. Liu Y. Q. Chen X.. (2020). Polyploidization of *Plumbago auriculata* Lam. in *vitro* and its characterization including cold tolerance. Plant Cell Tissue Organ Culture. 140, 315–325. doi: 10.1007/s11240-019-01729-w

[B24] Jin J. Cui H. M. Lv X. M. Yang Y. F. Wang Y. Lu X. Y. (2017). Exogenous CaCl_2_ reduces salt stress in sour jujube by reducing Na^+^ and increasing K^+^, Ca^2+^, and Mg^2+^ in different plant organs. J. Hortic. Sci. Biotechnol. 92, 98–106. doi: 10.1080/14620316.2016.1228435

[B25] Kobayashi H. Masaoka Y. Takahashi Y. (2007). Ability of salt glands in Rhodes grass (*Chloris gayana Kunth*) to secrete Na^+^ and K^+^ . Soil Sci. Plant Nutr. 53, 764–771. doi: 10.1111/j.1747-0765.2007.00192.x

[B26] Kong X. Q. Luo Z. Dong H. Z. Eneji A. E. Li W. J. (2016). H_2_O_2 and_ ABA signaling are responsible for the increased Na^+^ efflux and water uptake in *Gossypium hirsutum* L. roots in the non-saline side under non-uniform root zone salinity. J. Exp. Botany. 67, 2247–2261. doi: 10.1093/jxb/erw026 26862153

[B27] Li J. P. Fang Y. Liu Y. L. Zhang M. J. Liu Y. Zhao Y.. (2020). Exogenous melatonin enhances salt secretion from salt glands by upregulating the expression of ion transporter and vesicle transport genes in *Limonium bicolor* . BMC Plant Biol. 20, 493–493. doi: 10.1186/s12870-020-02703-x 33109099 PMC7590734

[B28] Lu Y. J. Li N. Y. Sun J. Hou P. C. Jing X. S. Zhu H. P.. (2013). Exogenous hydrogen peroxide, nitric oxide and calcium mediate root ion fluxes in two non-secretor mangrove species subjected to NaCl stress. Tree Physiol. 33, 81–95. doi: 10.1093/treephys/tps119 23264032

[B29] Ma L. Y. Zhang H. Sun L. R. Jiao Y. H. Zhang G. Z. Miao C.. (2012). NADPH oxidase AtrbohD and AtrbohF function in ROS-dependent regulation of Na^+^/K^+^ homeostasis in *Arabidopsis* under salt stress. J. Exp. Botany. 63, 305–317. doi: 10.1093/jxb/err280 21984648

[B30] Maeda Y. (2019). Effects of calcium application on the salt tolerance and sodium excretion from salt glands in zoysiagrass (*Zoysia japonica*). Grassland Sci. 65, 189–196. doi: 10.1111/grs.12234

[B31] Mahajan S. Pandey G. K. Tuteja N. (2008). Calcium- and salt-stress signaling in plants: shedding light on SOS pathway. Arch. Biochem. Biophys. 471, 146–158. doi: 10.1016/j.abb.2008.01.010 18241665

[B32] Mi P. Yuan F. Guo J. R. Han G. L. Wang B. S. (2021). Salt glands play a pivotal role in the salt resistance of four recretohalophyte *Limonium Mill.* Species. Plant Biol. 23, 1063–1073. doi: 10.1111/plb.13284 33969585

[B33] Mulaudzi T. Hendricks K. Mabiya T. Muthevhuli M. Ajayi R. F. Mayedwa N.. (2020). Calcium improves germination and growth of *Sorghum bicolor* seedlings under salt stress. Plants-Basel 9, 9060730. doi: 10.3390/plants9060730 PMC7356090 32531914

[B34] Munns R. Tester M. (2008). Mechanisms of salinity tolerance. Annu. Rev. Plant Biol. 59, 651–681. doi: 10.1146/annurev.arplant.59.032607.092911 18444910

[B35] Nomura H. Shiina T. (2014). Calcium signaling in plant endosymbiotic organelles: mechanism and role in physiology. Mol. Plant 7, 1094–1104. doi: 10.1093/mp/ssu020 24574521

[B36] Qiu Q. S. Guo Y. Dietrich M. A. Schumaker K. S. Zhu J. K. (2002). Regulation of SOS1, a plasma membrane Na^+^/H^+^ exchanger in *Arabidopsis thaliana*, by SOS2 and SOS3. Proc. Natl. Acad. Sci. United States Am. 99, 8436–8441. doi: 10.1073/pnas.122224699 PMC123085 12034882

[B37] Ruan C. J. da Silva J. A. T. Mopper S. Qin P. Lutts S. (2010). Halophyte improvement for a salinized world. Crit. Rev. Plant Sci. 29, 329–359. doi: 10.1080/07352689.2010.524517

[B38] Sanadhya P. Agarwal P. Khedia J. Agarwal P. K. (2015). A low-affinity K^+^ transporter *AlHKT2; 1* from recretohalophyte *Aeluropus lagopoides* confers salt tolerance in yeast. Mol. Biotechnol. 57, 489–498. doi: 10.1007/s12033-015-9842-9 25604033

[B39] Shabala S. (2019). Linking ploidy level with salinity tolerance: NADPH-dependent ‘ROS-Ca^2+^ Hub’ in the spotlight. J. Exp. Botany. 70, 1063–1067. doi: 10.1093/jxb/erz042 31222353 PMC6382325

[B40] Shabala S. Demidchik V. Shabala L. Cuin T. A. Smith S. J. Miller A. J.. (2006). Extracellular Ca^2+^ ameliorates NaCl-induced K^+^ loss from *Arabidopsis* root and leaf cells by controlling plasma membrane K^+^-permeable channels. J. Plant Physiol. 141, 1653–1665. doi: 10.1104/pp.106.082388 PMC1533937 16798942

[B41] Shabala S. Pottosin I. (2014). Regulation of potassium transport in plants under hostile conditions: implications for abiotic and biotic stress tolerance. J. Plant Physiol. 151, 257–279. doi: 10.1111/ppl.12165 24506225

[B42] Shuttleworth T. J. Hildebrandt J. P. (1999). Vertebrate salt glands: short- and long-term regulation of function. J. Exp. Zool. 183, 689–701. doi: 10.1002/(SICI)1097-010X(19990601)283:7<689::AID-JEZ7>3.0.CO;2-T 10222591

[B43] Shuttleworth T. J. Thompson J. L. (1989). Intracellular Ca^2+^ and inositol phosphates in avian nasal gland cells. Am. J. Physiol. 257, C1020–C1029. doi: 10.1152/ajpcell.1989.257.5.C1020 2596581

[B44] Sun J. Dai S. X. Wang R. G. Chen S. L. Li N. Y. Zhou X. Y.. (2009). Calcium mediates root K^+^/Na^+^ homeostasis in poplar species differing in salt tolerance. Tree Physiol. 29, 1175–1186. doi: 10.1093/treephys/tpp048 19638360

[B45] Sun J. Wang M. J. Ding M. Q. Deng S. R. Liu M. Q. Lu C. F.. (2010). H_2_O_2 and_ cytosolic Ca^2+^ signals triggered by the PM H^+^-coupled transport system mediate K^+^/Na^+^ homeostasis in NaCl-stressed *Populus euphratica* cells. Plant Cell Environ. 33, 943–958. doi: 10.1111/j.1365-3040.2010.02118.x 20082667

[B46] Terry B. R. Findlay G. P. Tyerman S. D. (1992). Direct effects of Ca^2+^-channel blockers on plasma membrane cation channels of *Amaranthus tricolor* protoplasts. J. Exp. Botany. 43, 7–1473. doi: 10.1093/jxb/43.11.1457

[B47] Wang W. L. Xing L. Xu K. Ji D. H. Xu Y. Chen C. S.. (2020). Salt stress-induced H_2_O_2 and_ Ca^2+^ mediate K^+^/Na^+^ homeostasis in *Pyropia haitanensis* . J. Appl. Phycol. 32, 4199–4210. doi: 10.1007/s10811-020-02284-0

[B48] Wegner L. H. De Boer A. H. Raschke K. (1994). Properties of the K^+^ inward rectifier in the plasma membrane of xylem parenchyma cells from barley roots: Effects of TEA^+^, Ca^2+^, Ba^2+^ and La^3+^ . J. Membrane Biol. 142, 363–379. doi: 10.1007/BF00233442 7707363

[B49] Yuan F. Chen M. Leng B. Y. Wang B. S. (2013). An efficient autofluorescence method for screening *Limonium bicolor* mutants for abnormal salt gland density and salt secretion. South Afr. J. Botany. 88, 110–117. doi: 10.1016/j.sajb.2013.06.007

[B50] Yuan F. Leng B. Y. Wang B. S. (2016). Progress in studying salt secretion from the salt glands in recretohalophytes: how do plants secrete salt? Frontiers in Plant Science 7, 977. doi: 10.3389/fpls.2016.00977 27446195 PMC4927796

[B51] Zhang W. D. Wang P. Bao Z. L. T. Ma Q. Duan L. J. Bao A. K.. (2017). *SOS1*, *HKT1*; 5, and *NHX1* synergistically modulate Na^+^ homeostasis in the halophytic grass *Puccinellia tenuiflora* . Frontiers in Plant Science 8, 576. doi: 10.3389/fpls.2017.00576 28450879 PMC5390037

[B52] Zherelova O. M. Grishchenko V. M. Chaylakhyan L. M. (1994). Blockers of Ca^2+^ channels in the plasmalemma of perfused *Characeae* cells. Comp. Biochem. Physiol. c-Toxicol. Pharmacol. 107, 475–480. doi: 10.1016/1367-8280(94)90079-5 8061954

[B53] Zhu J. K. (2016). Abiotic stress signaling and responses in plants. Cell 167, 313–324. doi: 10.1016/j.cell.2016.08.029 27716505 PMC5104190

